# Penicillin allergy de-labelling in primary care; correcting primary care records using a prescribing incentive scheme in Cornwall

**DOI:** 10.1093/jacamr/dlag156

**Published:** 2026-08-04

**Authors:** Neil Powell, Michael Wilcock, Paige Rickard, Stacie Tregonning, Chris Burgin, David Jenkins, Jo Walkers, Marco Motta

**Affiliations:** Pharmacy Department, Royal Cornwall Hospital Trust, Truro TR1 3LJ, UK; School of Biomedical Sciences, University of Plymouth, Plymouth PL4 8AA, UK; Pharmacy Department, Royal Cornwall Hospital Trust, Truro TR1 3LJ, UK; Medicines Optimisation Team, NHS Cornwall and IoS ICB, Bodmin PL31 2FR, UK; Medicines Optimisation Team, NHS Cornwall and IoS ICB, Bodmin PL31 2FR, UK; Medicines Optimisation Team, NHS Cornwall and IoS ICB, Bodmin PL31 2FR, UK; Helios Health Partnership, Helston Medical Centre, Trelawney Road, Helston TR13 8AU, UK; Helios Health Partnership, Helston Medical Centre, Trelawney Road, Helston TR13 8AU, UK; Medicines Optimisation Team, NHS Cornwall and IoS ICB, Bodmin PL31 2FR, UK

## Abstract

**Introduction:**

Penicillin allergy records are common and associated with several patient and health system harms. Using the local primary care prescribing and medicines optimization scheme (PMOS) for 2025/26 GP practices working across NHS Cornwall and Isles of Scilly integrated care board (ICB) in SW England, serving a population of 600 000, were financially incentivized to remove incorrect low-risk penicillin allergy (penA) records.

**Methods:**

Pharmacists from the ICB Medicines Optimisation Team, in partnership with an Antimicrobial Pharmacist at the local hospital developed the penicillin allergy de-label incentive scheme. GP records were searched for patients with a penA record and had subsequently received a prescription for any penicillin antibiotic. GP practice staff trained in penicillin allergy de-labelling (PADL) telephoned these patients to take a penA focused history, risk assess the history, confirm tolerance to the subsequent penicillin antibiotic and consent the patient to PADL. Successful completion of the PMOS scheme generated an incentive payment to practices.

**Results:**

In Cornwall, 50 of 55 (91%) practices engaged with the PMOS and returned data by 31 March 2026 on 3263 identified patients. Of these, 1627 (49.9%) were successfully contacted, of which 981 (60.3%) were eligible for PADL and 882 (54.2%) consented to PADL. The incentive scheme was delivered by practice-based pharmacy technicians, pharmacists and general practitioners.

**Conclusions:**

The study reports on the de-label of a large number of low-risk patients with penA across a primary care health community in the UK. This was possible due to education and training for a prescribing lead in each GP surgery coupled with financial incentive.

## Introduction

Penicillin allergy (penA) records are common with 8% of the UK general population reportedly having a penA record in their GP records.^1^ PenA records are associated with broader spectrum antibiotic prescribing and several associated negative patient and health system outcomes that include increased mortality and longer lengths of hospital stay.^2,3^ These associated harms are unnecessary because >90% of patients with a penA record can safely take penicillin antibiotics after formal allergy testing.^4^ In a UK primary care randomized controlled study, removal of penicillin allergy records by direct oral challenge (DOC) test or skin testing, called penA de-labelling (PADL), resulted in increased penicillin antibiotic prescribing, a corresponding reduction in broad spectrum antibiotic prescribing and reduced antibiotic prescribing overall.^5^ PADL is recognized by several international organizations and societies as a patient safety and antimicrobial stewardship priority.^6,7^

The lead author set up a hospital inpatient PADL patient pathway to de-label low-risk patients either after a DOC or on history alone if the history was consistent with a mild intolerance or a low-risk penA eligible for a DOC, but who had subsequently tolerated a penicillin and therefore eligible for direct de-label (DDL). Of those de-labelled, most (two-thirds) were de-labelled by DDL because the hospital prescribing system identified previous hospital administered penicillin, thus enabling DDL of patients with low-risk penA histories.^8^

Most patients with penA records are in primary care. Chan *et al.* used pharmacy staff in one GP practice in England to phone patients with a penA record and de-labelled those describing a reaction in keeping with drug intolerance. They successfully de-labelled 11 patients with patient satisfaction reported to be high.^9^

We aimed to use the local primary care prescribing and medicines optimization scheme (PMOS) for 2025–2026 to ask GP practices working across NHS Cornwall and Isles of Scilly integrated care board (ICB) in SW England, serving a population of 600 000, to identify low-risk penA patients and to remove incorrect penA records.

## Methods

Pharmacists from the ICB Medicines Optimisation Team, in partnership with the Lead Antimicrobial Pharmacist at the Royal Cornwall Hospital Trust—the principal secondary care provider for the Cornwall population—developed the PADL incentive scheme. The incentive scheme was approved through the ICB Medicines Optimisation Team governance structure and the PADL intervention was delivered in accordance with usual GP practice clinical governance frameworks. Designed for delivery by clinical staff within GP practices across Cornwall, the scheme was initially piloted by a small cohort of primary care pharmacists to test feasibility and ensure a robust and streamlined process prior to wider implementation. Results from the pilot were included in the final results. Successful completion of the PMOS scheme generated an incentive payment to practices, based on practice population size, and was costed at 15 pence (GB pounds sterling) per listed patient (proportional depending on the percentage of the list completed; see Appendix).

The process is described in the following subsections:

### Step 1: identifying eligible patients

GP electronic care records were searched, using electronic searches provided by the ICB, between April 2025 and March 2026 to identify patients with a penA record who had been prescribed a penicillin antibiotic after the allergy was recorded (see Appendix). Local GP care systems were either EMIS (originally Egton Medical Information Systems) or SystmOne (The Phoenix Partnership). Penicillin antibiotics included: amoxicillin, phenoxymethylpenicillin, co-amoxiclav, pivmecillinam, ampicillin, co-fluampicil and flucloxacillin.

### Step 2: patient assessment

Telephone consultation with the patient to take a penicillin allergy-focused history and record using the following questions adapted from Devchand *et al.*:^10^ which penicillin did you react to? Do you remember the details of the reaction? How many hours after taking the first dose did the reaction occur? How many years ago did this happen? How was the reaction managed, and what was the outcome? You were prescribed [antibiotic] on [date]. Did you take them, and if so, did you have any side effects?

The answers to the questions were used to assign the patient into a risk category using the antibiotic allergy assessment tool by Devchand *et al.*^10^ Patients were excluded if they could not provide a reliable history (i.e. they seemed confused, did not understand what was being asked of them or their account of their allergy history was contradictory). Patients categorized as high risk (red in the tool below) on the basis of their history were recorded as high risk and excluded. Patients who could not be contacted after three attempts were recorded as not contactable.

### Step 3: de-labelling process

Patients in the low-risk category (on the basis of history and evidence of subsequent tolerance of any penicillin) were de-labelled after counselling and obtaining informed consent.

### Step 4: updating patient records

With the patient’s consent, the penA record was removed from GP records. If possible, a follow-up message was to be sent to the patient to confirm the change. A template letter was included with the PMOS documentation; alternatively, practices could use their preferred electronic communication method. For non-dispensing patients, the patient’s usual community pharmacy was to be informed that the allergy label had been removed.

### Step 5: reporting

Practices were asked to submit the supplied log and reporting form by 31 March 2026.

Before the roll out of the scheme, all GP practice prescribing leads were invited to attend one of three education and training workshops delivered by the Consultant Pharmacist from the local hospital. The training material is in the Supplementary material (available as Supplementary data at *JAC-AMR* Online). In brief, it included a session on the importance of PADL, how to take a penA and risk assess a penA history and key counselling points.

## Results

Of the 55 practices in Cornwall, 50 (91%) practices engaged with the scheme and returned data by 31 March 2026 on 3263 identified patients. Healthcare workers reported to have delivered the incentive scheme included practice-based pharmacy technicians, pharmacists or general practitioners under the supervision of the prescribing lead for the GP surgery. The final cost to the ICB of the delivered PMOS was £74 515.85.

Of the 3263 identified patients, 1627 (49.89%) were successfully contacted. Of these, 981 (60.3%) were eligible for PADL and 882 (54.2%) consented to PADL (see Table 1).

**Table 1.** Patient demographics of both those identified via the search of GP records and those de-labelled

All identified eligible patients from the search		
Gender	Male (*n*)	1036
	Female (*n*)	1887
	Not recorded (*n*)	340
	Total (*n*)	3263
Age	Median (IQR)	57 (40–72)
	No age recorded (*n*)	192
Adults ≥18	*n*	2921
Paediatrics only <18	*n*	150
No age recorded	*n*	192
Age <18	median (IQR)	13 (9–15)
Unsuccessful contact with patients		1636/3263 (51.1%)
	3 unsuccessful contact attempts	371 (22.7%)
	2 unsuccessful contact attempts	82 (5.0%)
	1 unsuccessful contact attempts	408 (24.9%)
	No evidence of contact attempt made	259 (15.8%)
	Unknown contact attempts	516 (31.5%)
Successful contact made with patients	Yes (*n*; %)	1627/3263 (49.9%)
Eligible for de-label?	Yes (*n*; %)	981/1627 (60.3%)
	Consent to de-label (y)	882/981 (89.9%)
De-labelled cohort	Total	882
Gender	Male	313
	Female	500
	Not recorded	69
Age (total cohort)	Median (IQR)	58 (42–71)
Adults ≥18	*n*	858
Paediatrics only <18	*n*	24
Phenotype	Unknown reaction >10 years ago or family history only	287
	GI symptoms without other organ system symptoms (180 patients reported taking the subsequent penicillin, 7 had not taken it, 5 were unsure and for 28 this field was unrecorded).	220
	Diffuse rash or localized rash/swelling >10 years ago or unknown	198
	Childhood exanthem (unspecified)	88
	Not recorded	37
	Unknown reaction ≤10 years ago	21
	Mild neurological manifestation	15
	Diffuse rash or localized rash/swelling ≤10 years ago	10
	Mild hepatic enzyme derangement	3
	Mild renal impairment	1
	Urticaria	2
Reaction onset	Unknown (inc. blank)	724
	>24 hours	60
	2 to 6 hours	39
	6 to 24 hours	30
	<2 hours	29
How long ago?	>10 years ago	633
	Unknown or not recorded	160
	6 to 10 years ago	51
	1 to 5 years ago	35
	<1 year ago	3
Usual dispensing site informed of the updated penA status?	Yes	296
	No	152
	Not stated	434

IQR, interquartile range.

## Discussion

### Main findings

There was wide adoption of the PADL scheme, which was primarily delivered by GP practice-based pharmacists, under the supervision of a GP, with successful de-labelling of about half of those patients contacted.

### Comparison with the literature

A systematic review of primary care PADL studies in 2026 identified only two studies.^11^ Waldron *et al*. described their DOC study in an Australian primary care setting delivered by GPs trained to assesses severity of penA histories and successfully de-labelled 51 patients.^12^ Wittmer *et al.* describe their DOC in a Canadian sexual health clinic and de-labelled 26 patients via DOC.^13^ These studies differ from ours in that they both use DOC, whereas we use DDL and de-label relatively small numbers of patients. In the UK, Chan *et al.* described their primary care study using DDL in those patients’ reporting symptoms in keeping with intolerance, but again the numbers de-labelled were small (11 patients).

The common phenotypes de-labelled are in keeping with other PADL studies namely unknown reactions >10 years ago, diffuse rash >10 years ago, childhood exanthems and GI symptoms. There were two patients with urticaria, considered a higher risk phenotypes, both had subsequently tolerated the index penicillin.

### Strengths and limitations

This study reports the mobilization of a large multi-disciplinary primary care workforce across a health community to remove low-risk penA records demonstrating a way to de-label low-risk patients at scale and thereby reaching a wide population. Non-allergist delivered PADL is well embedded within the local hospital and awareness of the benefits of PADL are well known across the local health community, which may have contributed to the success of this PMOS thus limiting generalizability.

The impact of this initiative on future antibiotic prescribing both in primary care and secondary care were not evaluated and nor was the safety of this approach in that we do not know whether future penicillin exposure resulted in subsequent reactions. We also do not know the re-label rate, i.e. patients had their de-label status revoked.

### Further work and research

The numbers de-labelled represent only 1.84% of the estimated 48 000 patients in Cornwall with a penA record, assuming an 8% prevalence.^1^ How to safely de-label a greater proportion of patients with penA records in primary care needs further study. For a large number of patients, the contact attempts were unsuccessful, although we do not know why. Innovative methods for contacting patients should be explored to increase patient engagement. Further work is needed to ensure complete updating of allergy records across healthcare systems occurs, as we note community pharmacies were informed in only one-third of cases. Whether this intervention can be implemented in other ICBs needs further study. With the large number of GP practice staff now more aware of PADL and with PADL expertise in each GP practice, the future impact on de-labelling outside of the scheme needs to be determined. Gauging healthcare workers’ views on PADL to determine what lasting impact this scheme might have on AMS in primary care is also necessary. The impact of DDL on future prescribing of antibiotics and the safety of DDL delivered in primary care needs evaluation. Just over one in 10 patients eligible for PADL declined PADL; the reasons for which require further study.

### Conclusion

The study reports on the de-label of a large number of low-risk patients with penA across a primary care health community in the UK. This was possible due to education and training for a prescribing lead in each GP surgery coupled with financial incentive. The impact of such PADL initiatives on future prescribing and implementation into other regions in the UK needs further study.

## Supplementary Material

dlag156_Supplementary_Data
