# Supplementary material for: Penicillin allergy de-labelling in primary care; correcting primary care records using a prescribing incentive scheme in Cornwall
Source: JAC Antimicrob Resist. 2026 Aug 4;8(4):dlag156. doi: 10.1093/jacamr/dlag156 (PMC13435613; doi:10.1093/jacamr/dlag156)
Supplement: Supplementary file 1 [file dlag156_supplementary_data.zip › Appendix.docx]

**Antimicrobial resistance (AMR) project – removing inappropriate penicillin allergy labels.**

Practices will be paid up to 15p per listed patient for completing this project. A support document and searches are provided. Neil Powell, Consultant Pharmacist, Royal Cornwall Hospital Trust, will provide a bespoke training session to support. All clinicians involved in this project should attend this training or watch the recording before undertaking these conversations with patients.

The searches will identify patients that have a recorded penicillin allergy and received a penicillin antibiotic after this coding was added. Practices will need to discuss this with the patient and gain consent to remove the allergy coding if appropriate.

Practices will be paid for submitting a log (template provided) of their reviews by 31 March 2026. Practices should attempt to contact every patient (or their parent/guardian) identified by the search for a review.

It is appreciated that in some cases practices may not have the capacity to attempt to contact all the patients identified. Please leave all patients from the search results on the log and reporting form, regardless of whether any attempt was made to contact them, so that we can determine how many might remain eligible for de-labelling at the end of the scheme. Practices will then be paid a proportional amount based on the percentage of the list completed. For example, if a practice contacts 80% of their list identified, they will be paid 80% of the total payment available.
